# Small RNA Detection by *in Situ* Hybridization Methods

**DOI:** 10.3390/ijms160613259

**Published:** 2015-06-10

**Authors:** Martyna O. Urbanek, Anna U. Nawrocka, Wlodzimierz J. Krzyzosiak

**Affiliations:** Department of Molecular Biomedicine, Institute of Bioorganic Chemistry, Polish Academy of Sciences, Noskowskiego 12/14 Str., 61-704 Poznan, Poland; E-Mails: martyna.urbanek@ibch.poznan.pl (M.O.U.); anna.u.nawrocka@gmail.com (A.U.N.)

**Keywords:** short interfering RNA, Piwi-interacting RNA, LNA probe, rolling circle amplification, padlock probes, enzyme-labeled fluorescence signal amplification, TIRCA, PLA, tyramide signal amplification

## Abstract

Small noncoding RNAs perform multiple regulatory functions in cells, and their exogenous mimics are widely used in research and experimental therapies to interfere with target gene expression. MicroRNAs (miRNAs) are the most thoroughly investigated representatives of the small RNA family, which includes short interfering RNAs (siRNAs), PIWI-associated RNA (piRNAs), and others. Numerous methods have been adopted for the detection and characterization of small RNAs, which is challenging due to their short length and low level of expression. These include molecular biology methods such as real-time RT-PCR, northern blotting, hybridization to microarrays, cloning and sequencing, as well as single cell miRNA detection by microscopy with *in situ* hybridization (ISH). In this review, we focus on the ISH method, including its fluorescent version (FISH), and we present recent methodological advances that facilitated its successful adaptation for small RNA detection. We discuss relevant technical aspects as well as the advantages and limitations of ISH. We also refer to numerous applications of small RNA ISH in basic research and molecular diagnostics.

## 1. Introduction

There are several classes of small noncoding RNAs functioning in eukaryotic cells, which include, among others, 18–24 nt microRNAs (miRNAs), 21–22 nt short interfering RNAs (siRNAs), and 26–30 nt PIWI-associated RNAs (piRNAs). miRNAs are endogenous regulators of gene expression that primarily function at a posttranscriptional level, inhibiting mRNA translation with or without transcript degradation [1]. They also function in the cell nucleus and take part in gene transcription regulation. Exogenous miRNA mimics are used to compensate for pathological miRNA deficiency, and anti-miRNAs are delivered to cells to downregulate overexpressed miRNAs. Endogenous siRNAs that are present in both mammalian and plant cells take advantage of the miRNA pathway for their functioning. The same pathway is used by exogenous siRNAs, which are principal reagents of RNA interference technology. Both endogenous and exogenous siRNAs, due to full sequence complementarity to their targets, induce transcript cleavage. piRNAs, which do not use the miRNA pathway, function in germ cells to regulate transposon activity. Many difficulties in reliably determining cellular levels, establishing intracellular localization, and demonstrating regulatory interactions of these RNAs are associated with their small size.

Sensitive and highly specific small RNA detection is required not only for studying physiological processes regulated by these RNAs but also for better understanding relevant pathologies. Current research on small RNAs addresses numerous aspects of RNA biogenesis, localization, function and dysfunction. These aspects often need to be investigated in a single cell, with single molecule sensitivity, and spatial as well as temporal resolution. Physiologically, fluctuations of miRNA expression levels are observed during numerous cellular processes including cell division, maturation, and differentiation or after environment changes, e.g., drug treatment [2,3,4,5,6,7,8,9]. With regard to pathogenesis, changes in cellular levels of small RNAs can either be a cause or result of developing pathogenic processes. miRNA levels have been demonstrated to have a strong correlation with disease progression in cancer, cardiovascular disease, neurodegeneration, and numerous other pathologies [10,11,12,13,14,15,16]. Thus, miRNAs can be considered as diagnostic and prognostic disease markers.

Many novel approaches have been proposed to gain better insight into cellular levels and localization of miRNAs, which can also be used for detecting other types of small RNAs. The methods that take advantage of PCR-free signal amplification, nanotechnology and capillary electrophoresis have been recently reviewed by Tian and colleagues [17]. The only method that provides insight into both the level and localization in single cells is *in situ* hybridization (ISH) [18], which has increased considerably in importance in small RNA research over the last 10 years (Supplementary Figure S1). Depending on the detection method, ISH can be divided into chromogenic enzyme-based *in situ* hybridization and fluorescent *in situ* hybridization (FISH).

Multiple adaptations were included in standard ISH protocols for more efficient detection of small RNAs in cells, which we describe in detail in this review. We emphasize modifications at each step of the ISH protocols, probe design, cell fixation and permeabilization, hybridization, post-hybridization steps including washing, optional signal amplification and detection. Described protocol improvements provide better features, most of all higher sensitivity, specificity and resolution. We also present examples of small RNA ISH successful applications in different cell types and tissues, focusing on human and murine tissues.

## 2. Approaches for miRNA ISH

**Figure 1:**
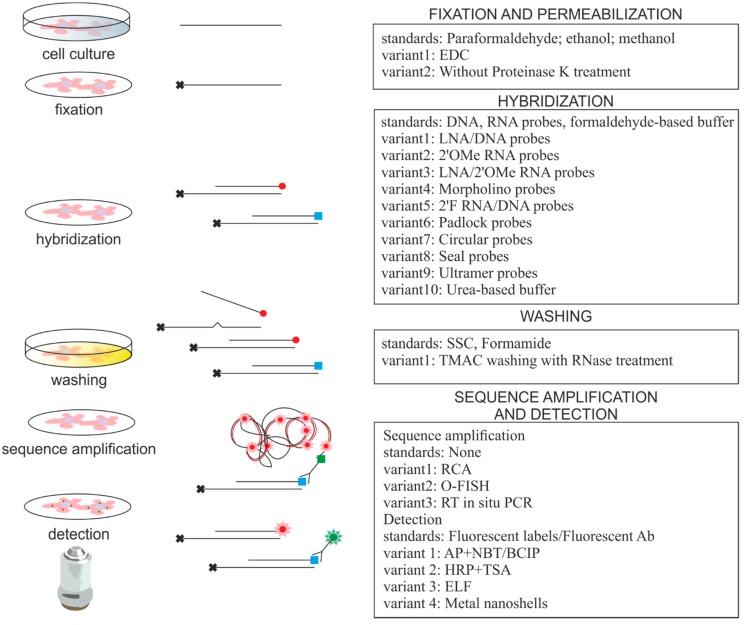
*In situ* hybridization protocols used for imaging of small RNAs. On the left are the steps of the ISH protocol on a cellular/tissue level, in the center are steps on a molecular level and on the right are modification variants of the ISH protocol used to detect small RNAs. Red and green dots represent fluorophores and squares represent non-fluorescent ligands. Black crosses indicate immobilization of miRNA.

miRNA ISH is exceptionally challenging because of miRNA features such as small size, sequence similarity among various miRNA family members and low tissue-specific or development-specific expression levels. The standard ISH protocol was modified to improve miRNA detection (Figure 1, Table 1) in various types of cell lines and tissues as well as whole embryos. Here, we describe these modifications with a focus on technical aspects and critically discuss these adaptations in the context of single molecule ISH and multi-miRNA detection.

**Table 1:** Variations in the critical steps of the small RNA *in situ* hybridization (ISH) protocol. All abbreviations are explained in the text.

miRNA ISH Protocol Variations	Advantages	Comments	References
LNA/DNA probes	High specificity and affinity	Golden standard in ISH, expensive	[5,9,19–36]
LNA/2′OMe RNA probes	Faster hybridization kinetics and ability to bind structured targets	Probes bind to blocking RNAs	[37,38]
RNA probes, TMAC washing, RNase A treatment	Single set of conditions for many probes, Tm for probe-target duplexes independent of GC composition, RNase A treatment decrease off-target binding	Applicable for multiplex analysis	[39,40]
2′F RNA/DNA probes	Increased hybridization efficiency, high selectivity	Applicable for high throughput analysis	[41]
Morpholino probes	High specificity and affinity	Hybridization is independent of salt concentration	[42]
DNA padlock probes, RCA	Up to single nucleotide specificity, RCA provides signal amplification	Applicable for detection of low abundant miRNA	[43]
DNA probes, PLA detection, RCA (O-FISH)	RCA as above	PLA originally used for protein detection	[44]
Circular DNA probes, RCA	Fast and efficient protocol, RCA as above	Applicable for multiplex analysis	[45]
Seal probes, RCA (TIRCA)	High specificity, decreased loss of miRNAs, RCA as above	Applicable for detection on single molecule level, low protocol temperature	[46]
Ultramer probes, RT *in situ* PCR	Signal amplification	Detects mature miRNAs only	[47]
Fluorescent metal nanoshell probes	Improved signal intensity and photostability	Improved optical properties of fluorophores, long lifetime emission signal	[36]
EDC fixation	Decreased loss of small RNAs, EDC immobilizes miRNA molecules	Important for low abundant miRNAs detection	[21,22,48,49]
NBT/BCIP detection system	Enhanced signal strength	Applicable for detection of low abundant miRNA	[6–8,50–53]
TSA detection system	Enhanced signal strength	Applicable for detection of low abundant miRNA	[22,25,31,32,38,48]
ELF detection system	High cellular resolution and signal strength	Applicable for detection of low abundant miRNA, single molecule detection, high photostability of precipitate, short exposure time	[41,49]

### 2.1. Probes

A very important step in an ISH experiment is probe design. Different probe types have different properties and detection options that enable selection of suitable solutions for many applications. These probes can be divided into two groups: linear probes directly labeled with fluorophore or ligand, and probes that enable sequence amplification.

#### 2.1.1. Directly Labeled Probes

**Figure 2:**
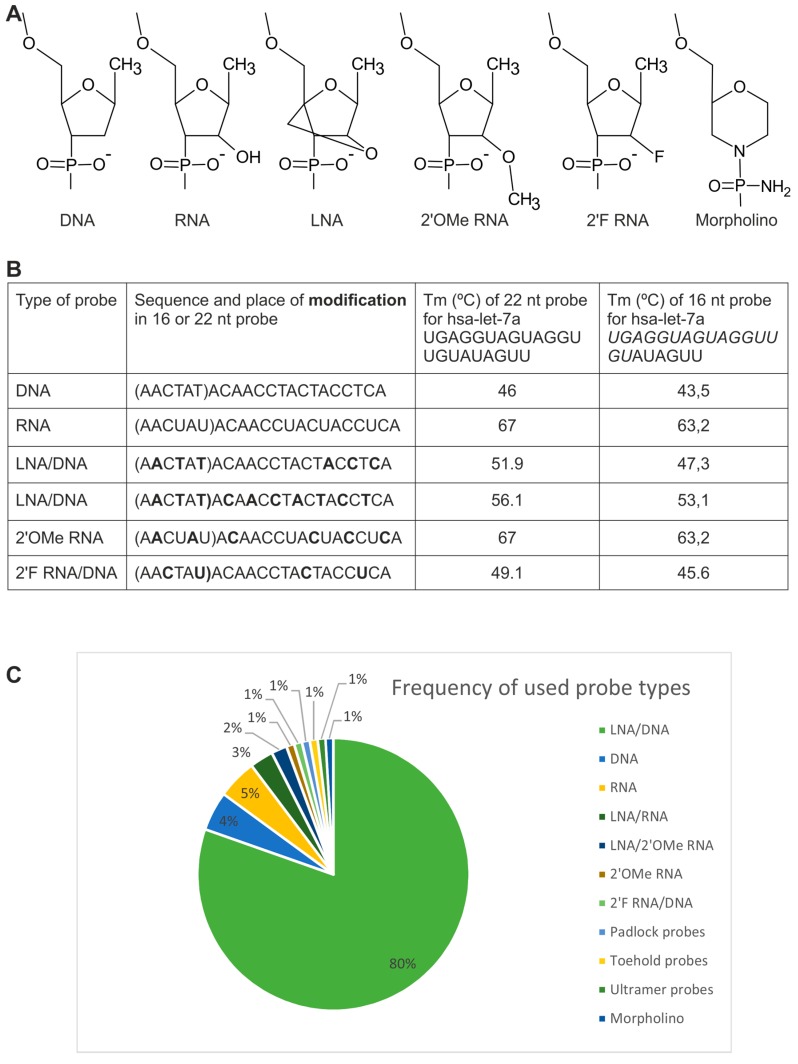
Types of nucleotide modifications used in small RNA ISH probes. (**A**) Chemical structure of modified nucleotides present in the probes; (**B**) Comparison of the melting temperature (Tm) of the 22 and 16 nt probes with different modifications (marked in bold) and RNA target (hsa-let-7a-1). Target sequence for shorter probe is marked in italic. The high melting temperature indicates strong binding with the target sequence. All Tm calculations were performed with IDT Technologies OligoAnalyzer 3.1; (**C**) Frequency of different types of probes used in small RNA ISH.

In standard ISH, probes composed of DNA or RNA nucleotides are commonly used. Unmodified DNA and RNA probes have relatively poor binding affinity to target sequences [54], and therefore several modifications have been proposed to improve their properties (Figure 2). First and most commonly applied was Locked Nucleic Acid (LNA) modification, which remains the gold standard in RNA FISH not only in small RNA detection. LNA nucleotides, referred to as “locked” RNA, have an additional bridge connecting 4′C and 2′O atoms. LNA nucleotides are incorporated into DNA probes, which leads to the formation of hybrid LNA/DNA probes. LNA/DNA probes have been shown to be highly beneficial in miRNA detection because of a short hybridization time, high efficiency, discriminatory power and a high melting temperature of the miRNA:probe complex. The minimal length of the LNA/DNA probe was determined to be 12 nucleotides [55] and these probes usually contain 30% LNA nucleotides. Besides their unquestionable advantages, these probes are expensive and can generate strong background signals resulting in a low signal-to-noise ratio for low abundant miRNA [56]. Therefore, other modifications were also proposed. These modifications include 2′fluoro-modified RNA (2′F RNA), morpholino, Zip Nucleic Acids (ZNA) [57], *N*,*N*-diethyl-4-(4-nitronaphthalen-1-ylazo)-phenylamine (ZEN) [58] and 2′O-Methyl (2′OMe) RNA modification. In comparison to DNA probes, 2′OMe RNA probes have faster hybridization kinetics and the ability to bind structured targets under standard conditions [59]. The combination of 2′OMe RNA and LNA modifications (in a 2:1 ratio) resulted in improved specificity and stability of the probe:RNA duplex in comparison to the LNA/DNA probe [38]. Specificity of the system may be further improved by shortening the probe length to 19 nt [59]. As the LNA/2′OMe RNA probe binds more strongly to yeast RNA or salmon sperm RNA used in ISH as a standard blocking agent, better results were obtained without these RNA blockers in the hybridization step [38]. 2′F RNA nucleotides incorporated in the DNA probes ensure increased binding to the target and better nuclease resistance [41]. Morpholino modifications, often applied to inhibit translation, modify splicing patterns of the primary transcript, or block miRNAs, were also used to detect miRNAs because of their high stability [42].

#### 2.1.2. Probes Used with the Sequence Amplification System

**Figure 3:**
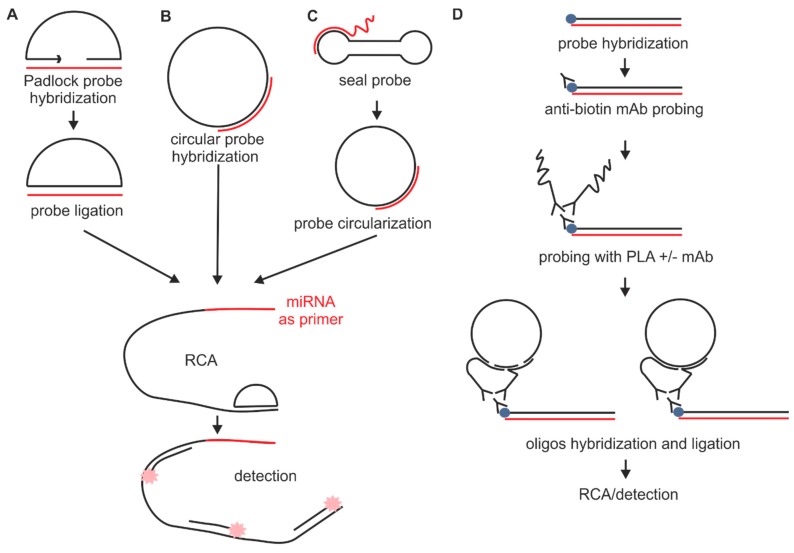

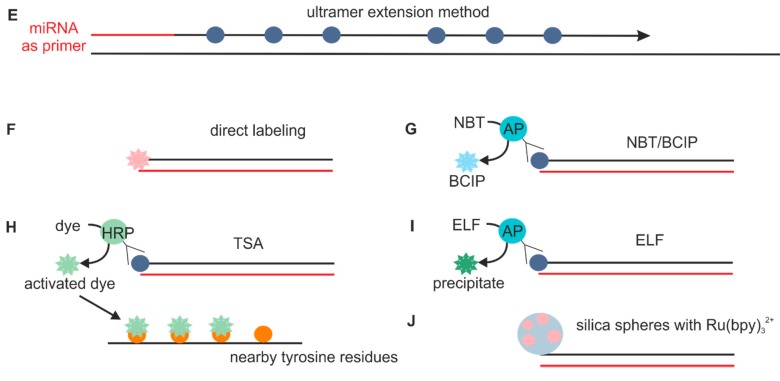
Sequence amplification and detection methods for small RNA ISH include (**A**) Padlock probes with RCA; (**B**) circular probes with RCA; (**C**) TIRCA; (**D**) O-FISH and (**E**) the ultramer extension method; Different methods for detection are also used for small RNAs including (**F**) direct labeling of probes (e.g., Cy3/fluorescein labeling); (**G**) NBT/BCIP; (**H**) TSA; (**I**) ELF and (**J**) silica spheres with Ru(bpy)_3_^2+^. miRNA is presented as red line and probe is shown as black line. Violet dot represents non-fluorescent ligand.

Several types of probes are combined with sequence amplification techniques to increase signal strength obtained from single miRNA molecules (Figure 3A–E). One example that enables sequence amplification is the use of padlock probes. These probes are successfully used to detect not only miRNAs but also mRNAs and DNA sequences. Padlock probes guarantee high specificity and sensitivity with single nucleotide discrimination [43]—what makes them applicable for allele-specific FISH [60]. Briefly, linear DNA probe after annealing to the specific sequence with 5′ and 3′ arms is circularized by DNA ligase. Circularization enables further signal amplification by rolling circle amplification (RCA) (Figure 3A). RCA uses miRNA molecule as a primer and elongates the sequence using circular probe as a template. Detection of RCA product is possible with the use of probes complementary to the sequence amplified on the template of the padlock probe central sequence. Similar types of probes, which are used in miRNA ISH also in combination with RCA, are circular DNA probes (Figure 3B). Circular probes are obtained *in vitro* with the use of padlock probes, ligation probes, and DNA ligase, and are then hybridized to the target sequence in cells as circular molecules [45].

Another type of probe used together with RCA is a seal probe with an adjustable toehold inside its loop (Figure 3). These probes have an ability to change their structure. The initial dumbbell shape of the probe is changed into a circular form when the target miRNAs bind into the toehold domain of the probe. As a result, a RCA reaction can be initiated. This new method, called toehold-initiated rolling circle amplification (TIRCA), is a combination of toehold-mediated strand displacement (TMSD) and RCA. The length of the toehold defines the stability of the probe and is the most important factor for detecting miRNAs with TIRCA. With increasing length of the toehold, the stability of the seal probe and selectivity of TIRCA decreases. There are many advantages in using TIRCA, such as reduced loss of miRNA molecules, because the detection process is conducted at a physiological temperature, short imaging time, and high sensitivity and specificity, which is even higher than in the case of a padlock probe based RCA reaction [46].

### 2.2. Fixation

The first step in the ISH protocol is cell fixation, which on one hand should sufficiently preserve the number and localization of small RNA molecules, but, on the other hand, is mild enough to preserve cellular domains crucial for detection, which is especially important for ISH combined with protein labeling. Prevention of miRNA loss during fixation is essential, especially for detecting low abundant miRNAs. Highly abundant miRNAs can be successfully detected using standard fixation protocols [19,20,23]. Significant improvement in miRNA recovery was shown by Tuschl and colleagues who used 1-ethyl-3-(3-dimethylaminopropyl) carbodiimide (EDC) to immobilize miRNA molecules via their 5′ end [21]. EDC fixation needs to be used in combination with traditional formaldehyde fixation. Improvement in the signal to noise ratio by addition of an EDC-crosslinking step was confirmed in several studies of miRNAs [21,22,42,45,48,61] (Table 1). Importantly, standard formalin-fixation and paraffin-embedding (FFPE) as well as cryopreservation of tissues seems to preserve microRNAs sufficiently [62].

### 2.3. Permeabilization

Permeabilization is used to improve cells and tissues penetration by the probe; however, permeabilization that is too strong can cause RNA loss from fixed material. Permeabilization is typically performed with the use of organic solvents including methanol or paraformaldehyde that are used also as fixatives, detergent like Saponin or Triton X-100, or proteinase. Briefly, organic solvents dissolve lipids from cell membranes. Some detergents, *i.e.*, Saponin, remove cholesterol from membranes in highly selective ways, but widely used Triton X-100 is not selective, which can lead to elimination of both proteins and lipids [63]. To reduce cellular RNA diffusion, treatment with proteinases and detergents was either limited or eliminated in some miRNA ISH studies [22,64]. The need for additional permeabilization decreases with the use of EDC fixation due to its auxiliary permeabilization activity. Long fixation of tissues also can decrease the need of permeabilization.

Some differences are observed in permeabilization techniques used in ISH of fixed tissues and cell lines. Typically, tissues have to undergo stronger permeabilization because of their lower accessibility to the probe; however, permeabilization conditions differ among different types of tissues. Tissues are typically permeabilized with Triton X-100, but Proteinase K is also used. However, permeabilization conditions for specific types of tissues, e.g., embryos or brain tissues, still need to be optimized [65]. Permeability of the tissue depends also on method of tissue preservation. Unfixed cryosections typically do not need permeabilization in contrary to fixed paraffin embedded sections. Cell lines are typically permeabilized with aceton, Triton X-100, Tween or overnight incubation with 70% ethanol. Fixation and permeabilization steps may be combined, e.g., by using a buffer containing paraformaldehyde and a short C-chain aliphatic carboxylic acid [66]. There are also several commercial buffers used for permeabilization [67].

Moreover, permeabilization conditions should be adjusted to the probe type and its length. Short linear probes used in miRNA ISH are able to easily enter cells and cellular compartments. Bigger, chemically modified probes or probes based on nanoshells would need stronger permeabilization treatment in comparison to short probes.

### 2.4. Hybridization

The hybridization step proceeds as in a standard ISH protocol. During hybridization, the optimal time of incubation and the temperature need to be experimentally determined because a calculated temperature for probe hybridization does not always give the best results. Typically the used hybridization buffer (HB) is the same as in ISH used for mRNA labeling; however, in some miRNA ISH experiments, HB was slightly modified. Important change in HB was the use of less toxic urea instead of 50% formamide [38]. In some specific experiments, it is desired to visualize more than one miRNA in a cell or tissue [22,38,68,69]. Multicolor ISH of miRNA may be performed sequentially or simultaneously. When simultaneous hybridization is performed and optimal hybridization temperatures for the probes differ, a suboptimal temperature must be chosen. When sequential hybridization is performed, hybridization can be performed at the optimal temperature for each probe [38].

### 2.5. Post-Hybridization Washing

The third step of an ISH protocol, which is crucial for specific detection of miRNA, is the washing step. Washing conditions need to be optimized to preserve the probe-target complex but at the same time eliminate off-target binding. One of the proposed improvements was changing the washing buffer composition to include tetramethylammonium chloride (TMAC). TMAC was used to compensate for the low specificity of RNA probes as highly stringent TMAC washing conditions decrease off-target binding [39,40]. Moreover, TMAC washing allows probes for different miRNAs to be detected in unchanged conditions, which can be used for *in situ* analyses with multiple miRNA probes. TMAC is also used in combination with RNase A to prevent off-target hybridization of RNA probes with mismatches [39]. In addition, it is important to adjust the washing temperature to provide specificity of binding while preserving the signal strength.

### 2.6. Sequence Amplification Methods

With the objective of single molecule detection, different types of sequence and signal amplification techniques have been used to provide better resolution (Figure 3). Enzymatic signal amplification methods, described in the detection section, are used mainly to image low-abundant miRNAs in tissues. The most commonly used system for sequence amplification is RCA [45,46]. The main advantages of RCA are better cellular resolution, super bright spots indicating miRNA and ultrahigh sensitivity [45]. RCA uses the free 3′ terminus of miRNA acting as the primer itself. mRNA and miRNA precursors do not give a positive signal as the 3′ end of these molecules does not hybridize to the probe and cannot be elongated during the process. The RCA process of sequence amplification is facilitated by DNA polymerase, resulting in the generation of thousands of amplified segments, which contain sequences complementary to the detection probes. The product of the RCA reaction is visualized with many fluorescently labeled detection probes.

The RCA method was combined with a proximity ligation assay (PLA) that led to development of a system named oligo-fluorescence *in situ* hybridization (O-FISH) (Figure 3D). The PLA technique was primarily developed to detect proteins with single molecule sensitivity. Briefly, biotin-labeled probe hybridizes to miRNA target and anti-biotin monoclonal antibodies detect the probe. The primary antibody is detected by two monoclonal antibodies conjugated with oligonucleotides. After secondary antibodies bind to the target, the antibody-conjugated oligonucleotides can hybridize to connector oligonucleotides due to their close proximity. Connector oligonucleotides are then ligated to circular DNA that can be used in RCA similar to the use of padlock probes. Oligonucleotides conjugated with antibodies serve as primers for RCA reaction [44].

Sequence amplification is also achieved by RT *in situ* PCR. This method, called the ultramer extension method, is based on the use of longer probes and miRNA acting as a primer, as in the RCA method. The probe contains sequences complementary to miRNA and a series of 20 nt sequences at the 5′ end. During *in situ* PCR, digoxigenin (DIG)-labeled nucleotides are incorporated to the PCR product to be further detected with antibodies [47].

### 2.7. Detection

Detection is the last, and a very important, step of miRNA ISH. In the first ISH experiments, radiolabeled probes were applied [18], but nonradioactive probes are currently used, giving information about the subcellular distribution of miRNA. In several experiments, probes directly labeled with fluorophores were used [5,24,26,70], but the obtained signal was not strong enough [45]. Weak emission signals, rapid photobleaching, strong photoblinking and a lifetime close to cellular autofluorescence are major shortcomings of organic fluorophores. Several signal enhancement methods have been proposed to overcome these difficulties. In the commonly used miRNA ISH technique, enzyme-based detection is used. Briefly, nonradioactive haptens, combined with probes, are detected by histochemical enzymatic reactions after application of enzyme-conjugated anti-hapten antibodies. Alkaline phosphatase (AP) is most commonly used with nitro blue tetrazolium/5-bromo-4-chloro-3-indolyl phosphate (NBT/BCIP) as a substrate [12,15,71,72,73]. The tyramide signal amplification system (TSA), or, in other words, the catalyzed reporter deposition method (CARD), is a similar approach. Early versions of the commercially available TSA/CARD system used streptavidin-biotin affinity for initial signal detection [68]. As endogenous biotin has been reported in many types of human tissue, currently LNA probes labeled with digoxigenin are mostly used in the hybridization step. In the TSA/CARD system, the horseradish peroxidase (HRP)-tagged anti-digoxigenin antibodies recognize digoxigenin moieties on probes. Next, HRP substrates, *i.e.*, cyanine 5 (Cy5), cyanine 3 (Cy3) or fluorescein-conjugated tyramides, are converted to highly reactive radicals by HRP and bind covalently to tyrosine residues located nearby [25]. The radicals are extremely short-lived, which prevents them from diffusing away from the site of synthesis, which would decrease the signal-to-noise ratio [48]. The TSA/CARD system improved the sensitivity up to 1000-fold compared to its early version [74].

Another detection method is enzyme-labeled fluorescence signal amplification (ELF). In short, pro-luminescent substrate cleavage is performed by phosphatase, followed by precipitation of a bright, yellow-green fluorescent product. The obtained precipitate is highly photostable and gives a 40 times brighter signal in comparison to probes directly labeled with fluorophores [41,75]. The dynamic range spans over three orders of magnitude, which means that 0 to 1000 copies of miRNA per cell can be quantified [49].

Nanotechnology was recently introduced in the field of miRNA ISH to improve the detection of small RNAs. Metal nanoshells composed of silica spheres with encapsulated Ru(bpy)_3_^2+^ complexes as cores and thin silver layers as shells have been used for the detection of low-abundant miRNAs. The metal nanoshells are based on near-field interactions between organic fluorophores and metal nanoparticles. The use of nanoshells resulted in overcoming difficulties specific to organic fluorophores, including reduced photoblinking, increased photostability and intensity, as well as an increased lifetime of the organic fluorophores in comparison to the lifetime of cellular autofluorescence [45]. However, the system needs to be optimized to provide better penetration of the nanoshells through the cell membrane, mobility in the cells as well as high specificity toward the miRNA target.

Commercial assays for the detection of several miRNAs using *in situ* hybridization and fluorescence *in situ* hybridization are also available [76,77]. A branched DNA system for signal amplification has been proposed to enable miRNA quantitative analysis [78]. The system was also shown to be efficient in the detection of exogenous siRNA molecules.

### 2.8. Specificity Controls

In RNA FISH, probes targeting different parts of mRNA are used to confirm probe specificity. This approach is not applicable in small RNA ISH due to miRNA short length. Thus, different controls need to be used to ensure probe specificity. In several experiments, probes complementary to miRNA analogs from different organism or even different kingdoms were used as negative control, e.g., for mammalian miRNA detection, probes against plant specific miRNA was used [53]. This approach may be applied only if miRNA analogs sufficiently differ in sequence. Another type of negative control is the use of probes against miRNA that is not expressed in the analyzed tissue (according to microarray or deep sequencing results) [79] or the use of scrambled probes [10,41,72,80]. The scrambled probe should be checked not only for specificity against miRNA population but also against the whole transcriptome. In some experiments, probes similar in sequence to miRNA-specific probes but containing two or three mismatches with the target sequence was used [38,40,55,79]. As a positive and negative control, miRNA-specific probes can be used to label different tissues that were shown to exhibit this miRNA expression or not, respectively [79].

To exclude false positive results caused by cells or tissue autofluorescence, control experiments without any probes are used [44]. Additionally, non-hybridization-based interactions are excluded by the treatment with unlabeled probes prior to hybridization with labeled probes [72]. Hybridization with probes that were already successfully tested can serve as positive controls for adequate experimental conditions and good RNA quality, e.g., snRNA U6 as a target is commonly used [10]. Interactions with DNA are excluded by DNase treatment and interactions with RNA are confirmed by RNase treatment prior to hybridization.

## 3. Applications of Small RNA ISH

Different ISH techniques are used to detect small RNAs depending on the required resolution (Table 2). Less precise enzymatic methods are typically used when low resolution is sufficient. When higher resolution is required, fluorescence-based techniques are used, which additionally enable studies of small RNA subcellular localization and function. Below, we briefly refer to the results of selected studies that were aimed at determining small RNA expression, subcellular localization and interactions and were obtained with methods differing in resolution.

Changes in miRNA expression levels have a significant impact on cells, tissues and whole organisms. These changes can be observed during physiological processes such as development [9,29], cell differentiation [33] and pathologies, e.g., cancer [27,28,30,31,32,34,35,36,32,34], cardiovascular [61,81,82] and neurodegenerative disorders [83,84,85,86,87,88,89]. Changes in miRNA levels were also observed during cell growth in specific conditions [57] or after drug treatment [3,4]. Observed changes rarely refer to single or a few miRNAs but more often to tens of miRNAs, and, therefore, multiplex ISH analyses are performed. In both research on physiological processes and various pathologies, the same ISH methods are applicable (Supplementary Table S1). The observed signal resolution depends mainly on the detection technique used. However, the critical step is probe design that distinguishes between closely related small RNA sequences, which is a very common problem for this class of RNAs.

Different miRNA ISH protocols are used in studies involving fixed tissues and cell lines. In tissue studies, typically LNA/DNA probes with a TSA or AP detection system is used (see Supplementary Table S1). In clinical diagnostics, where tissue samples are mainly used, the same methods apply. For example, in analysis of miRNA levels for diagnostic purposes in order to distinguish between various cancer types, LNA/DNA probes with TSA or AP detection systems were successfully used [19,20,22,90,91]. In cell line studies, higher resolution and sensitivity of imaging is often required, therefore, fluorescent detection as well as sequence and signal amplification techniques are applied. However, most commonly used LNA/DNA probes with direct or indirect fluorescent labeling remain the method of choice for the majority of applications.

### 3.1. Small RNAs in Tissues: Presence and Expression Levels

Enzyme-based methods, which are widely used in tissue research, have low resolution, which is compensated by high signal strength, low cost and simplicity of the methods. With use of enzyme-based techniques, changes in miRNA abundance are observed on a tissue level, where differences can be observed between different sections of the tissue. Mostly, LNA/DNA probes labeled with DIG or fluorescein are used and detected by AP or the TSA system. Co-localization with proteins is studied with the use of immunohistochemistry (IHC) or immunofluorescence (IF) to define the precise localization of RNAs in specific cell types [48,92].

For example, ISH was performed on mouse embryos to observe the influence of physiological cell-specific miRNA expression on mouse development and differentiation during embryogenesis [55], and similar studies were performed using chicken embryos [7] and zebrafish [9,29,93]. Tissue-specific expression levels of miRNAs were also observed in the brain in the context of synapse functions and plasticity [52,94,95] as well as drug addiction [3,4].

Tissue levels of miRNAs and cell type-specific miRNA expression are also widely studied in cancer [96,97,98,99]. Some miRNAs show a strong correlation with cancer progression [100,101], therefore, *in situ* analysis of miRNA abundance can be used as a diagnostic or progression marker [101,102]. Multiplex analysis of different miRNAs enables distinguishing between different types of cancers [22,103]. Examples of specific miRNAs that show high changes in cancerous cells that were imaged with ISH methods are miR-21 in colon adenocarcinomas and gliomas as well as pancreatic and breast cancer [99,100,104,105,106,107], miR-10b in pancreatic ductal adenocarcinoma [108] and miR-221 in breast cancer [109].

Methods enabling the localization of RNAs in specific cells in tissue samples were used also to image other types of small RNAs besides miRNAs. Most of the experiments used DIG-labeled LNA/DNA probes for hybridization to a target sequence, but various detection methods including AP and fluorophore-conjugated antibodies were used. Trans-acting siRNAs (ta-siRNAs) [110], different piRNA molecules and piRNA like small RNAs (pilRNAs) [111] were also localized successfully with the use of LNA/DNA probes. The same system was used to image miRNAs to confirm system reliability to detect all small RNAs [2]. Endogenous siRNAs present in plant cells were imaged simultaneously with miRNAs in plants using two-color labeling [112].

miRNA ISH based on enzymatic detection was also implemented in molecular diagnostics using tissue microarrays [99,113,114]. This technique enables fast detection of specific miRNAs in many tissue samples simultaneously. This high throughput analysis was performed with LNA/DNA probes and TSA labeling [64]. Automation in multiplex miRNA detection was also described with the use of the same detection system [69].

**Table 2:** Detection of small RNAs and biological material used with selected *in situ* hybridization (ISH) protocols.

Probe Type	Detection Method	Cell Lines	Cryosections	Paraffinic Tissue Sections	miRNA	siRNA	piRNA	Multiplex miRNA ISH	References
LNA/DNA probes	TSA	+	+	+	+	+	-	YES	[10,22,25,30,31,107,115]
LNA/DNA probes	AP	+	+	+	+	+	+	YES	[20,33,87,100,116]
LNA/DNA probes	direct or antibody-based fluorescent detection	+	−	−	+	−	+	YES	[94,96,103]
LNA/DNA probes	ELF	+	−	+	+	−	−	NO	[41,49]
DNA probes	RCA	+	−	−	+	−	−	NO	[44,45]

### 3.2. Small RNAs in Cells: Subcellular Localization

With increased resolution of *in situ* hybridization methods, it became possible to analyze subcellular localization of small RNAs. Because of the need for higher resolution, a number of studies on miRNA localization were performed with FISH methods. miRNAs have been found dispersed in the cytoplasm, nucleoplasm or localized in specific parts of cells. The ISH technique was used to observe miRNAs in specific parts of the nucleolus [5,24] and mitochondria [115]. To precisely localize small RNAs in the cell, protein markers of cellular compartments are usually labeled with IF [117] or with chimeric proteins fused to GFP [118]. Subcellular localization of small RNAs was investigated with the use of directly labeled Cy3-LNA/DNA probes [5,24], indirect labeling with fluorophore-conjugated antibody [119], or even double indirect antibody detection [120].

With the use of ISH techniques, miRNA biogenesis was also analyzed. Designing probes specific for the loop of miRNA precursors and probes specific for mature miRNAs enabled the comparison of levels and localization of both types of molecules [119]. RT *in situ* PCR and ultramer probes were used to discriminate between mature miRNAs and their precursors [47]. Padlock and circular probes with RCA detect only mature small RNAs; however, traditional probes are also used with the assumption that they are specific for mature miRNAs because of the secondary structure of their precursors.

Detection of RNA localization can also be useful to follow the cellular localization and fate of exogenous small RNAs. For example, miRNA ISH is used to monitor the degree of siRNA incorporation into cells and observe the cellular localization of siRNAs. Alternatively, siRNAs can be labeled directly to observe their cellular uptake; however, the label attachment can influence their localization and activity. It was shown that siRNAs can be efficiently detected with the use of LNA/DNA probes labeled with DIG. The detection systems used for this purpose include TSA [121,122] and AP [123,124]. siRNAs were also detected using 2′OMe RNA probes directly labeled with Cy3 [125]. The results of ISH were comparable with those obtained with direct labeling of small RNAs.

### 3.3. Small RNAs in Complexes: Co-Localization, Correlations and Interactions with DNA, RNA and Proteins

The combination of small RNA ISH with labeling of other RNAs, DNA and proteins can provide additional insight into small RNA functions. When such interactions are analyzed with *in situ* techniques, the obtained information is the existence or lack of co-localization, which does not necessarily mean direct interaction. Low resolution ISH combined with IF and IHC can show the correlation of cellular levels of miRNAs and proteins that are regulated by them. This functional dependence is observed as a negative feedback loop between miRNA and protein levels. Such correlations were observed in research concerning pluripotency, proliferation and differentiation [33] and in various cancers [97,126].

Co-localization of several miRNAs with other classes of biomolecules was performed with the use of fluorescent detection methods. For example, co-localization with U3 snoRNA, a marker of the granular component of the nucleolus, was conducted with the use of DIG-labeled LNA probes and fluorescently labeled antibodies [41]. Protein:miRNA co-localization was demonstrated for GW182 and miR-let7 in neurons with the use of DIG-labeled LNA probes [127].

To increase the resolution of such analyses, an improved fluorescent signal amplification system, called ELF, was used, which is claimed to be able to detect miRNAs on a single molecule level. The problem occurs when miRNAs are localized close to each other because they can be mistakenly interpreted as a single molecule. ELF is the only system that enabled single molecule detection and counting miRNA molecules while testing against Cy5-labeled, Texas red-labeled and quantum dot labeled secondary antibodies [49]. Several approaches based on RCA, which were also aimed at labeling single molecules, faced the same problem as ELF. These methods can be successfully applied to count small RNA granules in cells, however, without certainty that these granules contain single small RNA molecules.

## 4. Final Remarks and Future Perspectives

It is apparent from this review that a handful of *in situ* hybridization methods are now available for researchers, and the choice of the right method to use depends on the specific questions asked. Global miRNA expression analysis in tissues is typically performed with the help of chromogenic enzyme-based detection methods, but, for more precise small RNA localization studies, fluorescent imaging is more suitable. Sequence and signal amplification methods significantly increase the signal-to-noise ratio and enable the detection of low abundant RNAs. However, neither the RCA-based techniques nor ELF signal amplification methods are recommended when quantitative determination of small RNA levels are required. Therefore, choosing the right specific ISH variation is very important, and the intention of this review was to facilitate making such choices by providing some helpful comments on the existing methods and showing representative examples of their applications.

Despite the unquestionable success of the ISH adaptation for small RNA detection and subcellular localization, the various *in situ* hybridization techniques have their limitations. Their major shortcomings are the fact that they capture small RNAs in the cell at one point of time and are unable to distinguish between RNA functional and nonfunctional states. The latter may include RNA that is awaiting its cellular function or RNA stored for degradation. The only difference between the functionally active and inactive small RNAs might be the type of proteins or transcripts with which they interact while active or stored. The co-localization of miRNA with target mRNAs or Ago proteins would solve the problem partially. An important further insight could be provided by an analysis that captures the downstream effect of target transcript cleavage or degradation. Furthermore, there is no system available for live RNA imaging capable of labeling small RNAs [128]. The MS2 system was adapted for imaging miRNA primary precursors, called pri-miRNA [129,130] but not their processing products, mature miRNAs. Recently, the first attempt to monitor miRNAs in living cells was described [131].

Another important challenge is the ability of the ISH method for single molecule detection. Methods developed for single mRNA transcript detection [132,133,134] cannot be used for small RNA imaging because these methods are typically based on the use of multiple probes that target different regions of a single mRNA. Signal amplification techniques, which were adapted for small RNA analysis, enable the visualization of low abundant miRNAs; however, it has not yet been established whether these methods are capable of precise single molecule detection.

To summarize, several new approaches for the detection and analysis of small RNAs were proposed, enabling insight into the subcellular localization and co-localization of these molecules. The methods, in addition to their strong impact on the progress of basic miRNA research, also enabled the use of miRNAs as biomarkers for monitoring the progression of human diseases and following the outcomes of therapeutic treatments. With the available methods, the level and localization of small RNAs can be determined in single cells; however, temporal context and single molecule resolution is still needed, which remains a challenge for the development of future methods.

## Supplementary Materials

Supplementary materials can be accessed at: http://www.mdpi.com/1422-0067/16/06/13259/s1.
