# Supplementary material for: Small RNA Detection by in Situ Hybridization Methods
Source: Int J Mol Sci. 2015 Jun 10;16(6):13259–86. doi: 10.3390/ijms160613259 (PMC4490494; doi:10.3390/ijms160613259)
Supplement: Supplementary file 1 [file ijms-16-13259-s001.pdf]

Supplementary Information

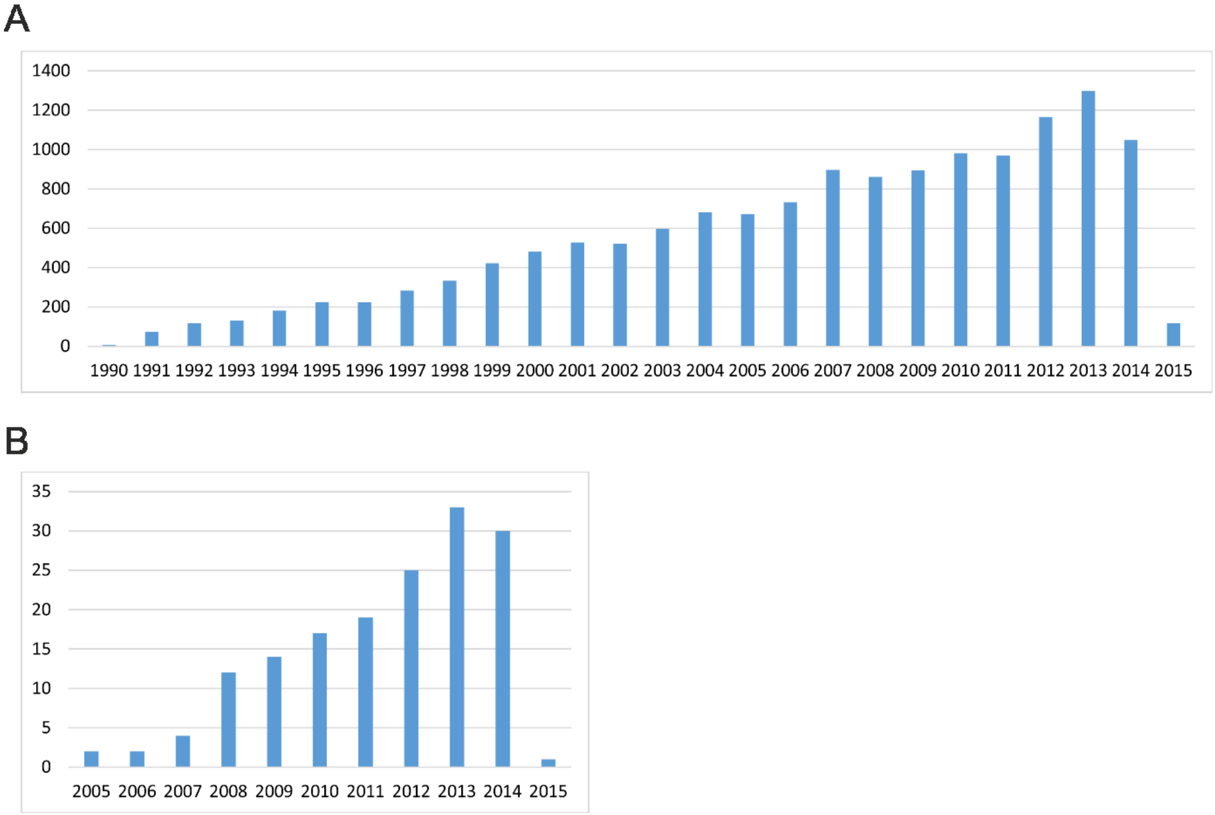

Figure S1. Numbers of PubMed publications found for (**A**) RNA FISH and (**B**) miRNA FISH search.

Table S1. Examples of small RNA ISH applications. Abbreviations: WISH-Whole mount *in situ* hybridization.

Visualized Small RNA	Cell Line/Tissue	Probe Type	Probe Tag	Signal Enhancement/ Detection Method	Comment	References
**Physiological miRNAs (Human, Mouse and Others)**						
mmu-miR-34b, mmu-miR-100, mmu-miR-465, mmu-miR-16	testis	LNA/DNA	DIG	AP+NBT/BCIP	protein labeling with IF	[2]
mmu-miR-200b	brain	LNA/DNA	DIG	HRP+TSA	cryosections	[135]
mmu-miR-718 mmu-miR-883a mmu-miR-883a	spermatogonia	LNA/DNA	DIG	AP+NBT/BCIP	cryosections, protein labeling with IF	[6]
dre-miR-124a, dre-miR122a, dre-miR-206, dre-miR-153, dre-pre-miR-183, dre-pre-miR-217, xtr-miR-124a, xtr-miR-1, 80 different mmu-miRNA	embryos	LNA/DNA	DIG	AP+NBT/BCIP	WISH	[55]
gga-miR-206, mmu-miR-206, xla-miR-206 and more	embryos	LNA/DNA	DIG	AP+NBT/BCIP	WISH, based on [53]	[7]
dre-miR-9a	embryos	LNA/DNA	DIG	AP+NBT/BCIP	protein labeling with IF, based on [136–138]	[139]
115 dre-miRNAs	embryos	LNA/DNA	DIG	AP+NBT/BCIP	WISH	[9]

Table S1. *Cont.*

Visualized Small RNA	Cell Line/Tissue	Probe Type	Probe Tag	Signal Enhancement/ Detection Method	Comment	References
			**Physiological miRNAs (Human, Mouse and Others)**			
mmu-miR-X, mmu-miR-Y, mmu-miR-410, mmu-miR-431, mmu-miR-500, mmu-miR-206	embryos	LNA/DNA	DIG	AP+NBT/BCIP	WISH, based on [9]	[53]
mmu-miR-140	embryos	LNA/DNA	DIG	AP+NBT/BCIP	WISH, based on [53]	[8]
mmu-miR-124a, mmu-miR-9, mmu-miR-92 and more	embryos, brain and eye	RNA	fluorescein	AP+NBT/BCIP	cryosections, based on [140]	[39]
111 gga-miRNAs	embryos	LNA/DNA	DIG	AP+NBT/BCIP	WISH, cryosections	[50]
gga-miR-146a, hsa-miR-146a,	DF1 fibroblasts, HeLa cells	DNA	biotin	RCA	-	[44]
mmu-miR-130a, mmu-miR-138, mmu-miR-195 and more	heart	LNA/2’OMe RNA	DIG or FITC	AP+NBT/BCIP, HRP+TSA	cryosections, FFPE, multiplex detection	[38]
mmu-miR-138	hippocampus	LNA/DNA	DIG	AP+NBT/BCIP, AP+Far Red	cryosections, protein labeling with IF	[51]
hsa-miR-124a, rno-miR-124a, mmu-miR-124a, mmu-miR-132 and more	brain	RNA	radiolabeled	AP+NBT/BCIP	cryosections, based on [140]	[40]

Table S1. *Cont.*

Visualized Small RNA	Cell Line/Tissue	Probe Type	Probe Tag	Signal Enhancement/ Detection Method	Comment	References
**Physiological miRNAs (Human, Mouse and Others)**						
mmu-miR-128, mmu-miR-200b, mmu-miR-195, mmu-miR-218 (microarray)	brain	LNA/DNA	DIG	HRP+TSA	cryosections	[113]
rno-miR-206, rno-pri-miR-206, rno-pre-miR-206, rno-let-7	L6 myoblasts	LNA/DNA	Cy3 or fluorescein	-	co-detection of miRNA, pri- and pre-miRNA, rRNA and protein (IF)	[5]
gga-miRNAs, ola-miRNas, dre-miRNAs, mmu-miRNAs	Chicken, medaka, zebrafish and mouse embryos	LNA/DNA	DIG	AP+NBT/BCIP	WISH, based on [50,55]	[141]
mmu-miR-124, mmu-miR-9, mmu-miR-410, mmu-miR-370, mmu-miR-26a, mmu-miR-26b and more	brain, heart and liver	LNA/DNA	DIG	AP+NBT/BCIP	protein labeling with IF or IHC	[21]
mmu-miR-100, mmu-miR-124a, mmu-miR-182, mmu-miR-96, mmu-miR-183	inner ear tissue (microarray)	LNA/DNA	DIG	AP+NBT/BCIP, AP+BM purple	WISH, based on [9,55]	[142]

Table S1. *Cont.*

Visualized Small RNA	Cell Line/Tissue	Probe Type	Probe Tag	Signal Enhancement/ Detection Method	Comment	References
**Physiological miRNAs (Human, Mouse and Others)**						
dme-pri-miR-10	embryos	RNA	DIG or biotin	HRP+TSA	WISH, co-detection of miRNAs and mRNA, multiplex detection	[68]
dme-pri-miR-124, dme-miR-91, dme-miR-279, dme-pri-miR-1	embryos	RNA	DIG	AP+NBT/BCIP, HRP+TSA	WISH, co-detection of pri-miRNA and mRNA, based on [68]	[143]
hsa-miR-145	embryonic stem cells	LNA/DNA	Cy3	-	-	[26]
rno-mir-124, rno-miR-181a, rno-let-7d	brain	LNA/DNA or RNA	DIG or radiolabeled	FITC or HRP	cryosections, FISH based on [79], ISH based on [144,145]	[3]
mmu-miR-9, mmu-miR-29c, mmu-miR-96, mmu-miR-124 and more	eye	LNA/DNA	DIG	AP+NBT/BCIP	cryosections, WISH, protein labeling with IF, based on [55]	[146]
rno-miR-125, rno-miR-199, rno-miR-1 and more	L6 myoblasts (microarray)	LNA/DNA	Cy3	-	protein labeling with IF, based on [5]	[24]
dre-miR-138	embryos	LNA/DNA	DIG	data not available	WISH, co-detection of mRNA and proteins (IHC)	[29]
mmu-miR-92a	vessels	LNA/DNA	DIG	AP+Far Red	based on [51]	[147]
mmu-miR-34a	Heart (microarray)	LNA/DNA	DIG	AP+Far Red	based on [147]	[116]

Table S1. *Cont.*

Visualized Small RNA	Cell Line/Tissue	Probe Type	Probe Tag	Signal Enhancement/ Detection Method	Comment	References
			**Physiological miRNAs (Human, Mouse and Others)**			
mmu-miR-24, mmu-miR-214, piR-7, piR-17005 and more	Spermatocytes (microarray)	DNA	Biotin	Avidin+FITC/Cy3	cryosections, protein labeling with IF	[148]
rno-miR-181c	neonatal ventricular myocytes (microarray)	LNA/DNA	DIG	HRP+TSA	co-localization of miRNAs in mitochondria, based on [79]	[115]
mmu-miR-9, mmu-pri-miR-9	brain	LNA/DNA	DIG	AP+NBT/BCIP	cryosections, WISH co- protein labeling with IHC, based on [149,150]	[33]
mmu-miR-132	hippocampal neurons	DNA	fluorescein	AP+NBT/BCIP	protein labeling with IHC, based on [151]	[52]
dre-miR-92b, dre-miR-124, dre-miR-9 and more	larvae	LNA/DNA	DIG	AP+NBT/BCIP	WISH, protein labeling with IHC, based on [9,55]	[93]
dre-miR-125b	embryos	LNA/DNA	DIG	AP+NBT/BCIP	WISH, based on [9]	[152]
hsa-miR-320, hsa-miR-107, hsa-miR-124a and more	brain	LNA/DNA	DIG	AP+NBT/BCIP	cryosections, based on [153,154]	[155]
rno-miR-212	Brain (microarray)	LNA/DNA	DIG	HRP+TSA	cryosections, protein labeling with IF, based on [79]	[4]
mmu-miR-132	brain	LNA/DNA	fluorescein	-	cryosections, protein labeling with IF, based on [73]	[94]

Table S1. *Cont.*

Visualized Small RNA	Cell Line/Tissue	Probe Type	Probe Tag	Signal Enhancement/ Detection Method	Comment	References
		**Physiological miRNAs (Human, Mouse and Others)**				
mmu-miR-30a, mmu-miR-30c, dre-miR-30a	embryonic liver, embryos (microarray)	LNA/DNA	DIG	HRP+TSA	protein labeling with IF, based on [79]	[156]
rno-pri-miR-132, rno-pri-miR-212, rno-miR-132, rno-miR-124a	Brain (microarray)	RNA or LNA/DNA	DIG	AP+NBT/BCIP, HRP+TSA	cryosections, based on [21,157]	[158]
hsa-pre-miR-302a, hsa-pre-let-7b, hsa-miR-365	myoblasts	LNA/DNA	DIG	anti-DIG fluorescein conjugated antibody	cryosections, localization of miRNAs in mitochondria, ISH and IF, based on [5]	[119]
		**miRNAs in cancer (human/mouse)**				
mmu-miR-124a, mmu-miR-1, mmu-miR-122, hsa-miR-128a, hsa-miR-21, mml-miR-128a, mml-miR-128b	brain, bladder, heart, hepatocytes, cerebellum	LNA/DNA	FITC or DIG	HRP+TSA	cryosections	[79]
hsa-miR-222, hsa-miR-223	hepatoma, hepatocytes	DNA	not applicable	RCA	-	[45]
hsa-miR-21	breast tumor tissue (microarray)	LNA/DNA	FAM	HRP+TSA	co-localization with proteins and other noncoding RNA (IHC), based on [159]	[107]
hsa-miR-21	breast tumor tissue	LNA/DNA	FAM	AP+NBT/BCIP	FFPE, protein labeling with IHC/FISH based on [20]	[106]

Table S1. *Cont.*

Visualized Small RNA	Cell Line/Tissue	Probe Type	Probe Tag	Signal Enhancement/ Detection Method	Comment	References
**miRNAs in cancer (human/mouse)**						
hsa-miR-21	breast tumor tissue	LNA/DNA	DIG	AP+NBT/BCIP	FFPE, protein labeling with IHC, based on [20]	[105]
hsa-miR-15a, hsa-miR-155	breast cancer cells, HeLa cells	LNA/DNA	DIG	ELF	detection of mRNA and miRNA	[49]
hsa-miR-146a	oral cancer tissue	LNA/DNA	DIG	HRP+TSA	FFPE	[25]
hsa-miR-221, hsa-miR-155, hsa-miR016	leiomyosarcoma malignant cells, malignant hepatocytes, heart and lung cells	LNA/DNA	DIG or biotin	AP+NBT/BCIP	FFPE, protein labeling with IHC	[160]
hsa-miR-21, hsa-miR-34a, hsa-miR-155, hsa-miR-24, hsa-miR-205, hsa-miR-126, hsa-miR-214, hsa-miR-375 and more	breast, colorectal, lung, pancreas, prostate carcinomas	LNA/DNA	FAM or biotin or BrdU or DIG	HRP+TSA	FFPE, co-localization with proteins and other noncoding RNA (IF)	[161]
hsa-miRNA-486	lung cancer cell lines	DNA, nanoshells	Cy3 or Cy5.5	-	-	[70]
hsa-miR-328, hsa-miR-221, hsa-miR-let-7c, hsa-miR-125b	colon and skin	LNA/DNA	DIG or biotin	AP+NBT/BCIP	FFPE, protein labeling with IHC	[73]
hsa-pri-mir-155	Hodgkin lymphoma and Burkitt lymphoma cells	RNA	DIG	AP+NBT/BCIP	FFPE, protein labeling with IF, based on [162]	[12]
hsa-miR-146a	oral cancer tissues	LNA/DNA	DIG or biotin	HRP+TSA	FFPE	[31]

Table S1. *Cont.*

Visualized Small RNA	Cell Line/Tissue	Probe Type	Probe Tag	Signal Enhancement/ Detection Method	Comment	References
			**miRNAs in cancer (human/mouse)**			
mmu-miR-205, mmu-miR-375	skin tumors (microarray)	LNA/DNA	Fluorescein or biotin	HRP+TSA	FFPE, co-localization of miRNAs with rRNA, protein labeling with IF, multiplex, based on [21]	[22]
hsa-miR-200a, hsa-miR-200b, hsas-miR-200c	stomach cancer cell lines, gastric mucosa and tumor samples (microarray)	LNA/DNA	biotin	cy3-avidin	protein labeling with IF and IHC	[96]
hsa-miR-21	colon, colorectal and rectal cancer tissues	LNA/DNA	DIG	AP+NBT/BCIP, HRP+TSA	FFPE	[20]
hsa-miR-21	tumor cell lines, colorectal tumor tissues	LNA/DNA	FITC	HRP+DAB	FFPE, protein labeling with IHC	[23]
hsa-miR-184, hsa-miR-146a	prostate cancer tissues (microarray)	LNA/DNA	fluorescein	-	protein labeling with IHC	[76]
hsa-miR-200c	bladder cancer (microarray)	LNA/DNA	DIG	AP+NBT/BCIP	FFPE, based on [159]	[34]
hsa-let-7b, hsa-miR-205	breast tumor tissue (microarray)	LNA/DNA	DIG	HRP+TSA	FFPE, protein labeling with IHC	[30]
hsa-miR-127, hsa-miR-154	bone marrow cells	LNA/DNA	DIG	FITC conjugated anti-DIG antibody	cryosections	[27]
hsa-miR-148b	Gastric cancer tissue	LNA/DNA	DIG	HRP+DAB	FFPE, cryosections	[32]
hsa-miR-221, hsa-miR-222	glioma tissues, fibroblast cell line	LNA/DNA	FITC	HRP+TSA	cryosections, protein labeling with IF, based on [23]	[36]
hsa-miR-138	ovarian cancer cell lines	LNA/RNA	biotin	HRP+TSA	protein labeling with IF	[35]
hsa-miR-205	prostate carcinoma cell lines (microarray)	LNA/DNA	DIG	AP+BM purple	-	[28]

Table S1. *Cont.*

Visualized Small RNA	Cell Line/Tissue	Probe Type	Probe Tag	Signal Enhancement/ Detection Method	Comment	References
**miRNAs in cancer (human/mouse)**						
hsa-miR-155, hsa-miR-16, hsa-miR-130, hsa-miR-221	colon cancer, placenta	LNA/DNA	DIG	AP+NBT/BCIP	FFPE, protein labeling with IHC	[47]
hsa-miR-155	lung cancer tissue (microarray)	LNA/DNA	DIG	AP+NBT/BCIP	based on [47]	[19]
hsa-miR-21	brain tumors	LNA/DNA	DIG	AP+NBT/BCIP	FFPE, based on [20,72]	[100]
mmu-miR-1, hsa-miR-124 and more	normal and cancer tissues	LNA/DNA	DIG	AP+NBT/BCIP	FFPE	[72]
hsa-miR-101	colon cancer cell lines and tumors	LNA/DNA	DIG	AP+NBT/BCIP, HRP+TSA	cryosections, protein labeling with IHC	[126]
hsa-miR-34a	prostate carcinoma tissue	LNA/DNA	DIG	AP+NBT/BCIP	FFPE	[98]
hsa-miR-155	breast cancer cell lines	LNA/DNA	DIG	data not available	cryosections, protein labeling with IHC, based on [13]	[97]
hsa-miR-21	colon tissue (microarray)	LNA/DNA	DIG	HRP+TSA	FFPE, based on [55]	[104]
hsa-miR-10b, hsa-miR-21, hsa-miR-155, hsa-miR-196a, hsa-miR-210	pancreatic cancer	LNA/DNA	FAM	HRP+TSA	FFPE, protein labeling with IHC	[108]
hsa-miR-205	malignant melanoma tissue (microarrays)	LNA/DNA	DIG	HRP+TSA	FFPE, protein labeling with IF, based [109]	[163]

Table S1. *Cont.*

Visualized Small RNA	Cell Line/Tissue	Probe Type	Probe Tag	Signal Enhancement/ Detection Method	Comment	References
**miRNAs in cancer (human/mouse)**						
mmu-miR-221, mmu-miR-21, mmu-miR-34a, mmu-miR-205, mmu-miR-92a	breast cancer (microarray)	LNA/DNA	DIG	HRP+TSA	FFPE, protein labeling with IF	[109]
hsa-miR-21	pancreatic cancer tissues (microarray)	LNA/DNA	DIG	AP+BM purple	FFPE, microarrays	[99]
hsa-miR-375, hsa-miR-146b	lung, esophageal cancer cell lines and tissues	2'F RNA/DNA	DIG	ELF	FFPE	[41]
mmu-miR-200a, mmu-miR-200b, mmu-miR-429	tumor samples	LNA/DNA	biotin	Cy3-avidin	based on [96]	[103]
hsa-miR-200a	ovarian cancer (microarray)	data not available	data not available	data not available	protein labeling with IHC	[164]
hsa-miR-708	ovarian cancer (microarray)	RNA/LNA	biotin	HPR+TSA	-	[114]
hsa-miR-21, hsa-miR-200c, hsa-miR-1246	cancer tissues	LNA/DNA	data not available	HRP+DAB	semi-automated method	[165]
hsa-let-7a	A549 cells	toehold probe	not applicable	TIRCA(FAM)	-	[46]
hsa-miR-126	cancer cell lines	LNA/DNA	biotin	AP+NBT/BCIP	protein labeling with IHC	[166]
hsa-miR-21	lymphoid tissues	LNA/DNA	fluorescein	HPR	FFPE, protein labeling with IHC, based on [161]	[167]
hsa-miR-34a	bladder tumor (microarray)	LNA/DNA	fluorescein	HPR	protein labeling with IHC, based on [161]	[168]

Table S1. *Cont.*

Visualized Small RNA	Cell Line/Tissue	Probe Type	Probe Tag	Signal Enhancement/ Detection Method	Comment	References
			**miRNAs in neurodegenerative diseases (human/mouse)**			
hsa-miR-206	muscle	LNA/DNA	DIG	FITC labeled anti-DIG antibody	cryosections	[11]
hsa-miR-107, hsa- miR-124	Brain (microarray)	LNA/DNA	DIG	AP+NBT/BCIP	based on [153]	[154]
mmu-miR-124, mmu-miR-16	hippocampus, central nervous system	LNA/DNA	DIG	AP+NBT/BCIP	protein labeling with IHC based on [51]	[14]
mmu-miR29a, mmu-miR-29b	brain	LNA/DNA	FITC	-	protein labeling with IF	[84]
hsa-miR-107, hsa-miR-124, hsa-miR-125b, hsa-miR-320	brain	LNA/DNA	DIG	AP+NBT/BCIP	cryosections, protein labeling with IHC, based on [153,154,169]	[170]
hsa-miR-338	leukocytes, serum, CSF and spinal cord	LNA/DNA	DIG	AP+NBT/BCIP	-	[87]
mmu-miR-9	spinal cord (microarray)	LNA/DNA	DIG	AP+NBT/BCIP	-	[86]
mmu-miR-29a, mmu-miR-29b, mmu-miR-219	brain	LNA/2’OMe RNA	FITC	HRP+TSA	cryosections, protein labeling with IHC	[37]
			**miRNAs in different diseases (Human/Mouse)**			
hsa-miR-377, mmu-miR-377	kidney (microarray)	LNA/DNA	DIG	AP+NBT/BCIP	based on [51]	[15]
hsa-miR-142, rma-miR-142	brain	LNA/DNA	DIG	HRP+TSA	FFPE, protein labeling with IF	[10]

Table S1. *Cont.*

Visualized Small RNA	Cell Line/Tissue	Probe Type	Probe Tag	Signal Enhancement/ Detection Method	Comment	References
			**Plants**			
tasiR-ARF, zma-miR-166	leaf	LNA/DNA	DIG	AP+NBT/BCIP	based on [171]	[110]
ath-miR-163, 45S siRNA, 5S siRNA	extracted nuclei	RNA	DIG or biotin	Alexa488-labeled anti-DIG antibody, Alexa543-labeled anti-biotin antibody	co-detection of siRNAs, protein labeling with IF	[112]
rma-miR-390, rma-miR-166, mmu-miR-122a	shoot apices	LNA/DNA	DIG	AP+NBT/BCIP	-	[172]
tasiR-ARF, rma-miR-390a, rma-miR-390b, rma-pri-miR-390	leaf	LNA/DNA	DIG	AP+NBT/BCIP	based on [171]	[173]
rma-miR-390, rma-miR-166, rma-ta-siARF	seedlings	LNA/DNA	DIG	AP+NBT/BCIP	-	[174]

Table S1. *Cont.*

Visualized Small RNA	Cell Line/Tissue	Probe Type	Probe Tag	Signal Enhancement/ Detection Method	Comment	References
**piRNAs and piRNA-like RNAs**						
rma-pil-RNAa, rma-pil-RNAb, rma-miR-206	cortex, prostate, epididymis, seminal-vesicles, testis	LNA/DNA	DIG	AP+NBT/BCIP	co-detection with transcripts	[111]
Transposonic 1, Transposonic 2, Sense Exonic, Anti-sense Exonic, Sense Intronic 1, Sense Intronic 2, Anti-sense Intronic, Repeat-associated1, Repeat-associated2, piRNA T4	spermatocytes, spermatids, spermatogonia, elongating spermatids	LNA/DNA	DIG	AP+NBT/BCIP	protein labeling with IF	[2]
piR-31, piR-80	testis, ovary	LNA/DNA	DIG	AP+NBT/BCIP	-	[71]
DQ541777, DQ540285, DQ705026, DQ719597	hippocampal neurons	LNA/DNA	DIG	Cy3-labeled anti-DIG antibody	protein labeling with IF	[175]
piR-49322	HeLa cells	LNA/DNA	DIG	AP	-	[176]
**Exogenous small RNAs**						
p54 siRNA	kidney sections	LNA/DNA	DIG	AP	siRNA tagged with Cy3, based on [153]	[123]
siRNA	skin sections	LNA/DNA	DIG	HRP+TSA	siRNA tagged with Cy3	[121]
scrambled siRNA	mouse skin	LNA/DNA	biotin	AP+ NBT/BCIP	RiboMap kit	[124]
sense and antisense strand of Ssb siRNA	mouse liver	LNA/DNA	DIG	HRP+TSA	protein labeling with IF	[122]
